# The prognostic significance of heart-type fatty acid binding protein in patients with stable coronary heart disease

**DOI:** 10.1038/s41598-018-32210-x

**Published:** 2018-09-26

**Authors:** Sing-Kong Ho, Yen-Wen Wu, Wei-Kung Tseng, Hsin-Bang Leu, Wei-Hsian Yin, Tsung-Hsien Lin, Kuan-Cheng Chang, Ji-Hung Wang, Hung-I Yeh, Chau-Chung Wu, Jaw-Wen Chen

**Affiliations:** 1 Cardiology Division of Cardiovascular Medical Center, Far Eastern Memorial Hospital, New Taipei City, Taiwan; 2 Cardiology Division, Department of Internal Medicine, Miaoli General Hospital, Ministry of Health and Welfare, Miaoli, Taiwan; 3 National Yang-Ming University School of Medicine, Taipei, Taiwan; 4 Department of Medical Imaging and Radiological Sciences, I-Shou University, Kaohsiung, Taiwan; 5 Division of Cardiology, Department of Internal Medicine, E-Da Hospital, Kaohsiung, Taiwan; 6 Institute of Clinical Medicine and Cardiovascular Research Center, National Yang-Ming University, Taipei, Taiwan; 7 Divison of Cardiology, Department of Medicine, Taipei Veterans General Hospital, Taipei, Taiwan; 8 Division of Cardiology, Heart Center, Cheng-Hsin General Hospital, and School of Medicine, National Yang-Ming University, Taipei, Taiwan; 9 Division of Cardiology, Department of Internal Medicine, Kaohsiung Medical University Hospital and Kaohsiung Medical University, Kaohsiung, Taiwan; 10 Division of Cardiovascular Medicine, China Medical University Hospital, Taichung, Taiwan; 11 Graduate Institute of Biomedical Sciences, China Medical University, Taichung, Taiwan; 12 Department of Cardiology, Buddhist Tzu-Chi General Hospital, Tzu-Chi University, Hualien, Taiwan; 13 Cardiovascular Division, Department of Internal Medicine, MacKay Memorial Hospital, Mackay Medical College, New Taipei City, Taiwan; 14 Division of Cardiology, Department of Internal Medicine, National Taiwan University Hospital and National Taiwan University College of Medicine, Taipei, Taiwan; 15 Graduate Institute of Medical Education & Bioethics, College of Medicine, National Taiwan University, Taipei, Taiwan

## Abstract

To investigate the prognostic value of heart-type fatty acid binding protein (H-FABP) in patients with stable coronary heart disease (SCHD). A total of 1,071 patients with SCHD were prospectively enrolled in this Taiwan multicenter registry study, followed for 24 months. The cut-off value of H-FABP, 4.143 ng/mL, was determined using receiver operating characteristic curves. The primary cardiovascular (CV) outcome was composite CV events, defined as cardiovascular or cerebrovascular death, myocardial infarction (MI), stroke, angina related-hospitalization, PAOD-related hospitalization and heart failure. Secondary outcomes included CV or cerebrovascular death, nonfatal MI, nonfatal stroke, and acute heart failure-related hospitalization. We found that the high H-FABP group had more than a two-fold higher rate of primary CV outcomes than the low H-FABP group (32.36% vs. 15.78%, *p* < 0.001). Eleven patients (4.82%) of the high H-FABP group died during the 24 months of follow-up, compared to only one patient (0.12%) in the low H-FABP group. The acute heart failure-related hospitalization rate was also significantly higher in the high H-FABP group (3.5% vs. 0.95%, *p* < 0.005). The results remained significant after adjusting for baseline covariates. In conclusion, H-FABP was an independent predictor for CV outcomes in the patients with SCHD, mainly in CV death and acute heart failure-related hospitalization.

## Introduction

Ischemic heart disease and stroke have been the leading causes of death globally in the past decades, and the mortality rate from these diseases is gradually increasing. In addition to traditional cardiovascular (CV) risk factors such as smoking, type 2 diabetes mellitus (T2DM), hypertension (HTN) and dyslipidemia, researchers have investigated potential novel biomarkers, for instance, copeptin^1^, pentraxin-3^2^ and heart-type fatty acid binding protein (H-FABP) to predict the clinical course and CV outcomes. In particular, H-FABP has been widely studied in patients with acute coronary syndrome (ACS), and it has been suggested to increase diagnostic sensitivity and possibly predict long-term survival^3^.

H-FABP is a human protein that is encoded by the fatty acid binding protein 3 (FABP3) gene and is located on chromosome 1p32-p35. It is a cytoplasmic protein which was first isolated from ischemic rat hearts in 1988, and was identified as being released from injured myocardium^4,5^. Associations between H-FABP and ACS^6–8^, acute kidney injury^9^, post-cardiac surgery^10^, acute pulmonary embolism^11^, acute ischemic stroke^12^, severe sepsis^13^, acute heart failure^14^, hypothyroidism^15^ and hyperthyroidism^16^ have been reported over the past decades. On the other hand, H-FABP has also been used to assess perioperative cardiac risk^17,18^. However, the prognostic implication of H-FABP in patients with stable coronary heart disease (SCHD) is unknown. The aim of this study was to investigate the prognostic value of H-FABP in CV outcomes in patients with SCHD.

## Results

### Patients

A total of 1,072 SCHD patients from the National Taiwan Biosignature Research (NTBR) cohort study were enrolled and followed for 24 months or until a CV event. At 24 months, 207 cardiovascular events had occurred, including 12 CV deaths, 24 nonfatal myocardial infarction (MI), 6 nonfatal strokes and 16 acute heart failure-related hospitalizations (Table 1).

**Table 1:** Cardiovascular Events in 24 months.

Events	Number of case (%)
Total CV events	207
CV or cerebrovascular death	12 (5.8%)
Nonfatal myocardial infarction	24 (12%)
Nonfatal stroke	6 (3%)
Angina-related hospitalization	132 (64%)
PAOD-related hospitalization	14 (7%)
Heart failure Acute heart failure-related hospitalization Aortic dissection Brady- or tachyarrhythmia	19 (9.2%) 16 (7.7%) 1 (0.5%) 2 (1%)

CV = cardiovascular, MI = myocardial infarction, PAOD = peripheral arterial occlusive disease.

**Figure 1:**
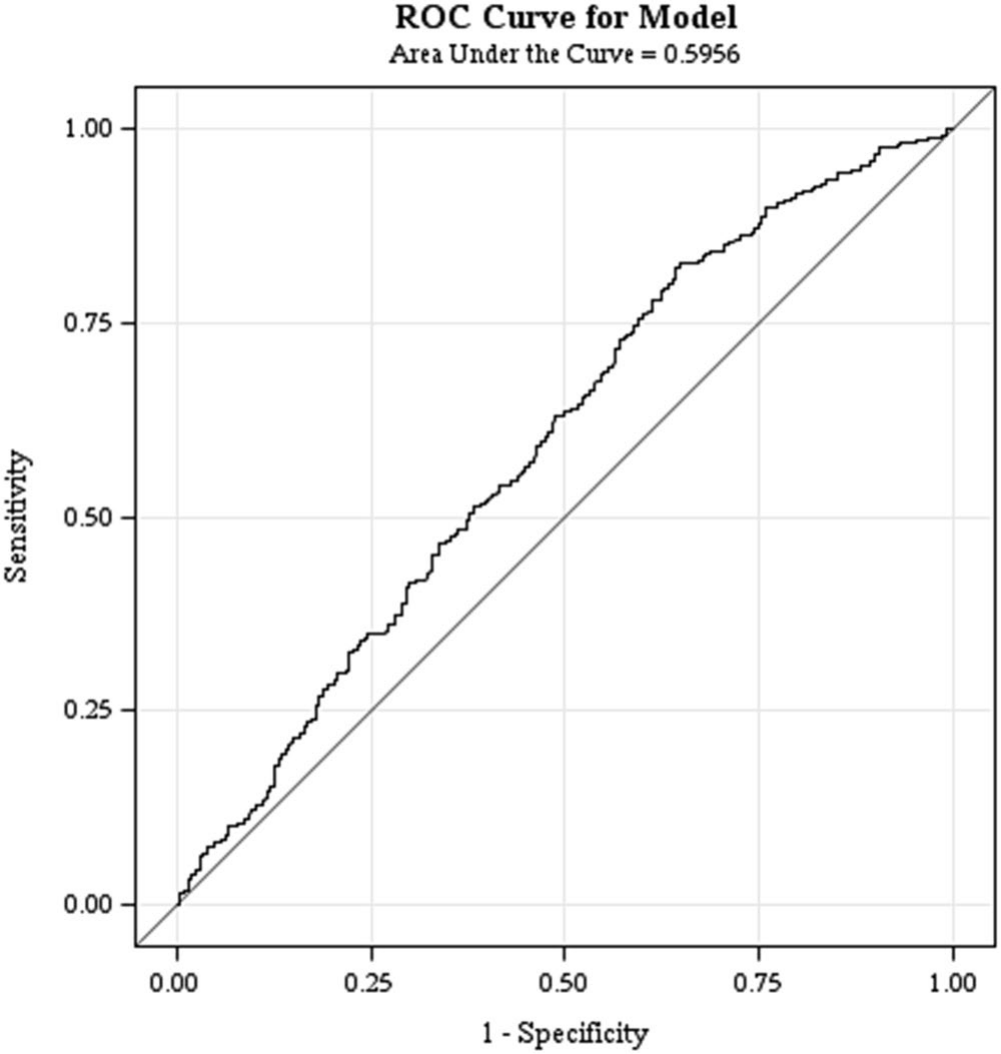
Receiver operating characteristic curve (ROC) analysis plot with area under the curve, sensitivity and specificity of H-FABP in prediction of total cardiovascular events.

The cut-off value of H-FABP (4.143 ng/mL) was determined by receiver operating characteristic curves (ROC) curve analysis (Fig. 1) between the patients with and without CV events from the blood sample obtained at enrollment. The baseline characteristics revealed that the patients with a high level of H-FABP had higher rates of HTN, but lower rate of family history of premature coronary artery disease (CAD). Except for a lower level of serum high-density lipoprotein cholesterol (HDL-C), patients with a high level of H-FABP had significantly higher blood glucose, systolic blood pressure (SBP), serum creatinine, high sensitivity C-reactive protein (hs-CRP) and N-terminal pro-brain natriuretic peptide (NT-proBNP) than those with a low level of H-FABP (Table 2).

**Table 2:** Baseline characteristics of patients with stable coronary heart disease.

	Total	(%)	H-FABP < 4.143 ng/mL		H-FABP ≧ 4.143 ng/mL		*p*
	n		n	(%)	n	(%)	
Male gender	1071	913 (85.25%)	843	718 (85.17%)	228	195 (85.53%)	0.894
Hypertension	1071	698 (65.17%)	843	531 (62.99%)	228	167 (73.25%)	0.004
Diabetes	1071	408 (38.1%)	843	283 (33.57%)	228	125 (54.82%)	<0.001
Smoking	1071	603 (56.3%)	843	482 (57.18%)	228	121 (53.07%)	0.267
Family history of premature CAD	1071	246 (22.97%)	843	213 (25.27%)	228	33 (14.47%)	0.001
Previous stroke	1071	28 (2.61%)	843	22 (2.61%)	228	6 (2.63%)	0.985
1-vessel disease	1071	596 (55.65%)	843	482 (57.18%)	228	114 (50%)	0.138
2-vessel disease	1071	165 (15.41%)	843	124 (14.71%)	228	41 (17.98%)	0.118
3-vessel disease	1071	21 (1.96%)	843	16 (1.9%)	228	5 (2.19%)	0.699
		**Median (IQRs)**		**Median (IQRs)**		**Median (IQRs)**	
Age, year	1071	64.9 (57.2–74.3)	843	63.3 (56.5–71.6)	228	72.5 (62.2-81.0)	<0.001
BMI (kg/m^2^)	1070	25.9 (23.7-28.3)	842	26.0 (23.7–28.4)	228	25.6 (23.4–28.4)	0.555
Systolic BP, mmHg	1071	130 (119–114)	843	130 (119–140)	228	131 (120–147)	0.016
Diastolic BP, mmHg	1071	74 (66–83)	843	75 (67–83)	228	73 (65–83)	0.112
Glucose, mg/dL	1065	106 (95–131)	839	105 (94–126)	226	114 (97–143)	0.002
Hemoglobin, g/dL	1019	13.6 (12.4–14.9)	796	14.0 (12.8–15.1)	223	12.4 (11.0–13.7)	<0.001
LDL-C, mg/dL	1066	90 (73–111)	841	90 (74–112)	225	91 (72–108)	0.322
HDL-C, mg/dL	1065	40 (35–48)	840	41 (35–48)	225	38 (33–45)	0.007
Serum creatinine, mg/dL	1066	1.03 (0.87–1.28)	839	0.98 (0.83–1.14)	227	1.50 (1.19–2.36)	<0.001
eGFR, mL/min/1.73 m^2^	1066	74 (59–90)	839	79 (66–95)	227	68 (28-63)	<0.001
hs-CRP, mg/dL	779	0.14 (0.07–0.31)	611	0.13 (0.07–0.27)	168	0.22 (0.10–0.58)	0.004
NT-pro BNP, pg/mL	1071	171 (66–460)	843	141 (58–367)	228	334 (109–880)	0.001

Results are expressed as percentage or medians (IQRs).

BMI = body mass index; BP = blood pressure; CAD = coronary artery disease; eGFR = estimated glomerular filtration rate; HDL-C = high-density lipoprotein -cholesterol; LDL-C = low-density lipoprotein-cholesterol; hs-CRP = high sensitivity C-reactive protein; NT-pro BNP = N-terminal pro-brain natriuretic peptide.

### Primary outcomes

**Figure 2:**
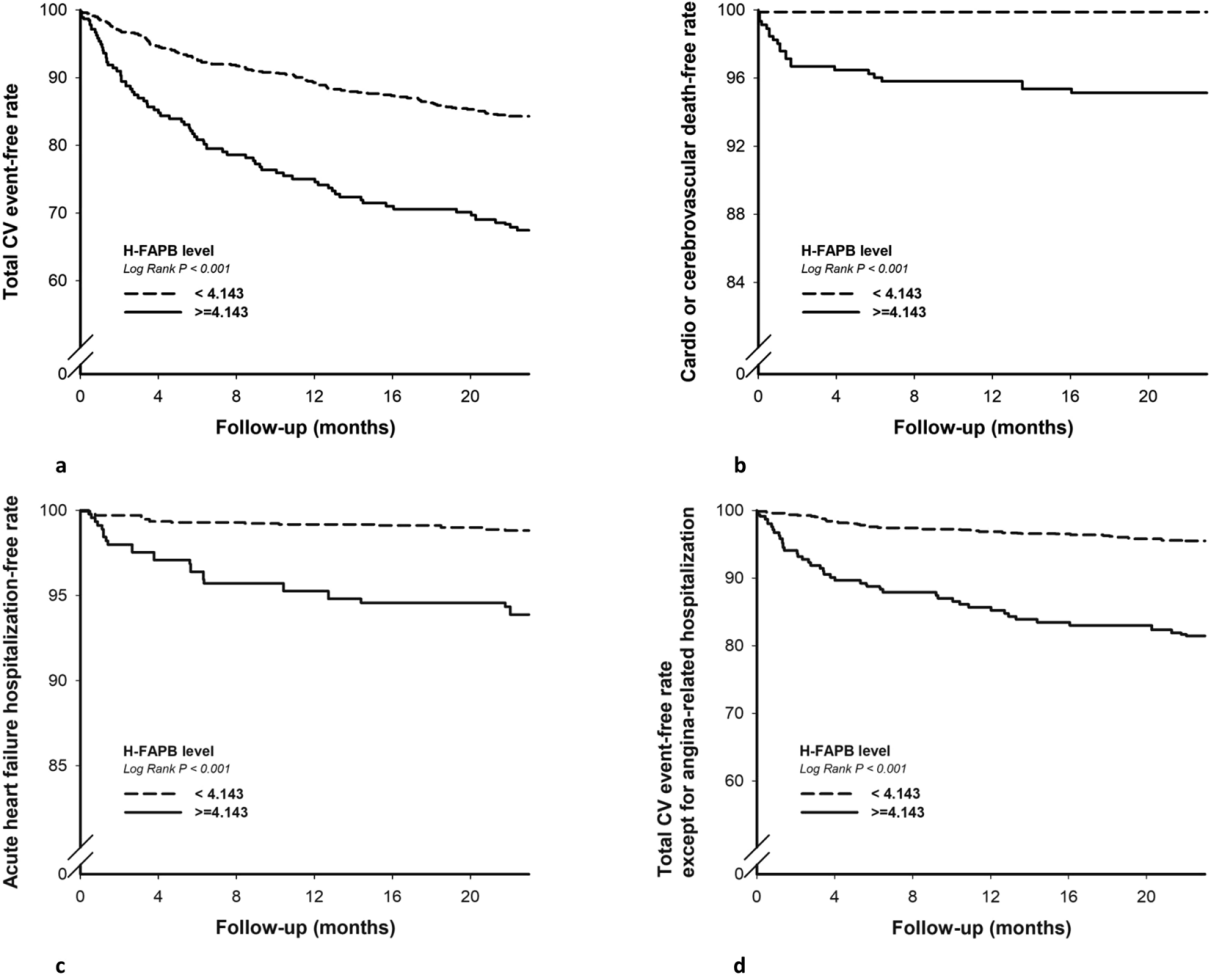
Kaplan–Meier survival curves analysis showing total cardiovascular (CV) event-free rate (**a**), CV or cerebrovascular death-free rate (**b**), acute heart failure hospitalization-free rate (**c**), and total CV event-free rate except for angina-related hospitalization (**d**) in patients with serum H-FABP ≧ 4.143 ng/mL and H-FABP < 4.143 ng/mL (all *p* < 0.001).

After 24 months of follow up, the high H-FABP group had more than a two-fold higher rate of primary CV events than the low H-FABP group (32.36% vs. 15.78%, *p* < 0.001) (Table 3). The Kaplan-Meier curves of the two groups were significantly separated from the beginning of the study to 24 months (Fig. 2a).

**Table 3:** Clinical outcomes in 24 months.

	All (n = 1,071)	H-FABP < 4.143 ng/mL, (n = 843)	H-FABP ≧ 4.143 ng/mL, (n = 228)	*p*
**Primary outcome**				
Total CV events, n (%)	207 (19.33%)	133 (15.78%)	74 (32.46%)	<0.001
**Secondary outcome**				
CV or cerebrovascular death, n (%)	12 (1.12%)	1 (0.12%)	11 (4.82%)	<0.001
Nonfatal myocardial infarction, n (%)	24 (2.24%)	16 (1.9%)	8 (3.51%)	0.145
Nonfatal stroke, n (%)	6 (0.56%)	3 (0.36%)	3 (1.32%)	0.085
Acute heart failure-related hospitalization, n (%)	16 (1.49%)	8 (0.95%)	8 (3.51%)	0.005
Total CV events except for angina-related hospitalization, n (%)	80 (7.47%)	38 (4.51%)	42(18.00%)	<0.001

CV = cardiovascular.

### Secondary outcomes

A total of 11 deaths (4.82%) occurred in the high H-FABP group, compared with only one (0.12%) in the low H-FABP group (Table 3 and Fig. 2b). In addition, the high H-FABP group had a significantly higher rate of acute heart failure-related hospitalizations (3.5%) compared to the low H-FABP group (0.95%) (Table 3 and Fig. 2c). Although statistically non-significant, there was also a trend of higher rate of nonfatal MI and nonfatal stroke in the high H-FABP group (Table 3). There were 80 patients with total CV events except for “angina-related hospitalization”, 38 patients in H-FABP group, 42 patients in H-FABP group (4.51% vs 32.46%, *p* < 0.001). The difference between these two groups remained significant (Fig. 2d).

In multivariate Cox proportional hazards analysis adjusted for age, sex, body mass index (BMI), serum creatinine, estimated glomerular filtration rate (eGFR), HDL-C, hemoglobin (Hb), blood glucose, hs-CRP, NT-proBNP, SBP, smoking, family history of premature CAD, history of hypertension and diabetes mellitus, high H-FABP level was still an independent prognostic risk factor for CV events (HR 2.93, 95% CI 1.95–4.394, *p* < 0.001). In addition, a high level of H-FABP also predicted CV death (HR 22.89, 95% CI 2.16–242.55, *p* = 0.009) and acute heart failure-related hospitalizations (HR 5.16, 95% CI 1.096–24.324, *p* = 0.038) in the 24-month follow-up period, even after adjusted for other covariates (Table 4).

**Table 4:** Univariate and multivariable logistic Cox-proportional regression analysis models for clinical outcomes.

	Univariate*		Multivariate**	
	HR (95% CI)	*p*	HR (95% CI)	*p*
**Primary outcome**				
Total CV events	2.35 (1.77–3.13)	<0.001	2.93 (1.95–4.39)	<0.001
**Secondary outcome**				
CV or cerebrovascular death	41.75 (5.39–323386)	0.004	22.89 (2.16–242.55)	0.009
Nonfatal myocardial infarction	1.96 (0.84–4.58)	0.120	2.62 (0.80–8.59)	0.112
Nonfatal stroke	3.91 (0.79–19.35)	0.085	1.56 (0.06–38.62)	0.786
Acute heart failure related hospitalization	3.90 (1.46–10.39)	0.007	5.16 (1.10–24.32)	0.038

CV = cardiovascular.

*Non-adjusting.

**Adjusting for significant variables in univariate analysis, which including age, gender, body mass index, serum creatinine, estimated glomerular filtration rate, high-density lipoprotein, low-density lipoprotein, hemoglobin, fasting glucose, high sensitivity C-reactive protein, N-terminal pro-brain natriuretic peptide, systolic blood pressure, history of hypertension, smoking and diabetes, family history of premature CAD.

## Discussion

This study is the first prospective cohort study to demonstrate that a higher serum H-FABP level (≧4.143 ng/mL) is an independent predictor for CV events, particularly for cardio- and cerebrovascular death and acute heart failure-related hospitalizations in patients with SCHD. Our result was concordant with the Takahata study^19^, which also found that H-FABP level was increased in association with greater numbers of cardiovascular risk factors. In addition, Takahata study noted higher H-FABP level was an independent risk factor for all-cause and cardiovascular deaths in 3,503 subjects who participated in a community-based health checkup in a 7-year follow-up.

The early diagnosis of acute MI is still challenging for emergency physicians despite the wide application of myoglobin and high-sensitivity cardiac troponin (cTn) in emergency rooms, because the elevation of most myocardial injury serum markers are delayed by at least 2–4 hours after an ischemic insult. In 2000, an experimental study of ligation of the left main coronary artery in mice demonstrated that the concentration of H-FABP at 4 hours could be used to stratify MI compared to cTn at 48 hours^20^. In addition, Okamoto *et al*. reported that H-FABP is more sensitive than myoglobin and creatinine kinase isoenzyme MB for the diagnosis of acute MI in the early phase^21^. In 2006, O’Donoghue *et al*. reported an association between an elevated level of H-FABP and increased risks of death and major cardiac events in patients with ACS^22^. Collinson *et al*.^23^ compared the diagnostic performances of cTn-I, H-FABP and copeptin in low-risk patients presenting with chest pain. The authors concluded that cTn-I remained the best single test, with the incremental diagnostic sensitivity of serum H-FABP, but not copeptin. Furthermore, a recent dobutamine stress echocardiography (DSE) study reported significantly increased levels of serum H-FABP at 1 hour in the presence of DSE-induced ischemia, in contrast to DSE negative group, whose serum H-FABP remained unchanged before and 1 hour after the test^24^. However, in a study that was expected H-FABP to increase during exercise stress testing (EST), serum H-FABP tended to decline statistically significant from the basal level to 3 hours after the EST^25^. A recent systemic review of H-FABP in ACS found marked heterogeneity in the prognostic impact of H-FABP between studies, reflecting differences in sampling times and the population at risk. Hence, it may not be possible to routinely use H-FABP as a prognostic marker in patients with suspected ACS^26^.

Wunderlich *et al*. were the first to report that an early elevation of serum H-FABP and brain type fatty acid binding protein (B-FABP) concentration were significantly associated with the severity of neurological deficits and functional outcomes in patients after an acute ischemic stroke^12^. The peak levels of H-FABP and B-FABP occur 2 to 3 hours after an event and remain elevated for up to 120 hours. In addition, a high level of H-FABP is associated with large infarctions on brain computed tomography. Another investigation of 41 patients with acute stroke (31 with ischemic stroke, 10 with intracerebral hemorrhage) demonstrated that serum H-FABP and ischemic-modified albumin (IMA) levels increased within 4.5 hours^27^. Nonetheless, An *et al*. reported that H-FABP was not an independent marker in patients with ischemic stroke, and thus that its clinical usefulness is limited^28^. In the current study, we demonstrated the prognostic value of H-FABP in CV events in patients with SCHD after successful treatment, but that it had limited value in the prediction of nonfatal MI and ischemic stroke. In this study, although statistically non-significant, there was also a trend of higher rate of nonfatal MI and nonfatal stroke in the high H-FABP group.

The relationship between H-FABP and heart failure was first reported in the early 2000s, when the concentration of H-FABP was positively correlated with the concentration of BNP in patients with acute deterioration of heart failure^29^. Later, Setsuka *et al*.^30^ reported that H-FABP was present in the activation of tumor necrosis factor (TNF) and the Fas ligand system. This suggested a pathophysiological role of cardiomyocyte necrosis and/or apoptosis in patients with worsening heart failure. Moreover, Hoffmann *et al*.^14^ investigated H-FABP in acute heart failure, and found that additional H-FABP measurements improved the diagnostic specificity and positive predictive value of NT-proBNP tests. In addition, their patients in the highest H-FABP quartile had significantly higher rates of all-cause mortality (HR 2.1–2.5; *p* = 0.04) and risk of re-hospitalization for acute heart failure at 5 years (HR 2.8–8.3, *p* = 0.001). Our study also demonstrated that the SCHD patients with high H-FABP level had a higher risk for acute heart failure-related hospitalizations at 24 months.

There are several limitations of this study. First, even though the criteria for patient enrollment and the protocol for clinical follow-up were clearly defined, selection bias arising from clinical profiles, investigator participation and treatment adherence by the patients could not be completely excluded^31^. Second, this is a hospital based rather than a community-based study, and this design was potentially limited by geographic variations such as environmental exposure to risk factors of CV disease^32^. Third, all the patients were stable during enrollment and followed up regularly for clinical events in the out-patient clinics of the medical centers. Their medications may have been adjusted by the specific cardiologists during follow-up. Thus, the potential effects of different cardiovascular drugs on clinical outcomes could not be well addressed^33^. Fourth, the very few cases of the each secondary event category, insufficient statistical power of predictive value of H-FABP could be derived from the multivariate analyses.

In conclusion, H-FABP was an independent predictor for total CV events in the patients with SCHD at 24 months, mainly for CV and cerebrovascular deaths and acute heart failure-related hospitalization.

## Methods

### Study population

This NTBR was a prospective cohort study of patients with SCHD (aged ≧ 20 years) from nine medical centers in Taiwan^31^. At enrollment, all of the participants had undergone a percutaneous coronary intervention at least once and had been stable on medical treatment for at least 1 month. The exclusion criteria included hospitalization for any CV event within 3 months, and those unable or unwilling to be followed up during the following 1 year period. Specific clinical outcomes including all-cause, cardiovascular, cerebrovascular mortalities, and CV-related hospitalizations were confirmed using the Health and Welfare Data Science Center (HWDC) of Taiwan.

This study complied with the Declaration of Helsinki and was approved by the appropriate Health Authorities, independent Ethics Committees, and Institutional Review Boards (IRB) in each hospital as well as the Joint IRB Ethics Committee Review Board in Taiwan. All of the patients agreed to participate and signed the informed consent form.

### Baseline clinical and biomarker data collection

After enrollment, data were prospectively collected by physicians and nurses whenever feasible. Baseline characteristics included sex, age, HTN, T2DM, hyperlipidemia, smoking, family history of premature CAD, BMI, number of stenotic coronary arteries, and biochemical data including renal function, lipid profile at enrollment in each hospital were recorded. Hs-CRP was performed automatically with chemiluminescent immunoassay methods, on a Beckman Coulter DXC 800 immunoassay platform (Beckman Coulter, Inc. CA, USA). NT-pro BNP and H-FABP were measured manually on EMD Millipore’s MILLIPLEX MAP Human CVD 1 Magnetic Bead kit (Millipore, Inc. MO, USA).

### Clinical follow-up

Questionnaire and blood samples were obtained from the patients every 3 months in the first year and every 6 months thereafter for a total of 24 months. The primary CV outcome was composite CV events, defined as cardiovascular or cerebrovascular death, MI, stroke, angina-related hospitalization, PAOD-related hospitalization and heart failure. Heart failure was a composite of acute heart failure-related hospitalization, syncope, cardiopulmonary resuscitation, bradyarrhythmia, supraventricular tachyarrhythmia, ventricular arrhythmia, permanent pacemaker implantation and aortic dissection. The secondary outcomes included CV or cerebrovascular death, nonfatal MI, nonfatal stroke, and acute heart failure-related hospitalization.

### Statistical analysis

The cut-off value of H-FABP was determined using ROC curve analysis between the patients with and without CV events from the blood sample obtained at enrollment. Baseline characteristics and CV outcomes were compared between the patients with high and low levels of H-FABP.

Results are expressed as median (interquartile ranges [IQRs]) for continuous variables, and qualitative variables are expressed in absolute frequencies (number of patients) and relative frequencies (percentage). Comparisons of continuous variables between groups were performed using ANOVA or Mann-Whitney *U* tests. The primary and secondary outcomes were described as overall percentages and expressed as means of proportions with a 95% confidence interval (CI). The Kaplan-Meier method was used to calculate events and survival rates. Hazard ratios (HRs) for the regression of Cox proportional hazards were used, along with the corresponding standard error, 95% CI, and *p* value. Independent baseline variables with a *p* value < 0.05 in the univariate analysis were included in the multivariate analysis. In all the tests, the two-tailed alpha significance level was 0.05. In addition, *p* values were reported up to three decimals, while those below 0.001 were reported as *p* < 0.001.

## Electronic supplementary material

Supplementary Information
