# Author Correction: The prognostic significance of heart-type fatty acid binding protein in patients with stable coronary heart disease

**DOI:** 10.1038/s41598-018-36737-x

**Published:** 2019-03-13

**Authors:** Sing-Kong Ho, Yen-Wen Wu, Wei-Kung Tseng, Hsin-Bang Leu, Wei-Hsian Yin, Tsung-Hsien Lin, Kuan-Cheng Chang, Ji-Hung Wang, Hung-I Yeh, Chau-Chung Wu, Jaw-Wen Chen

**Affiliations:** 1 Cardiology Division of Cardiovascular Medical Center, Far Eastern Memorial Hospital, New Taipei City, Taiwan; 2 Cardiology Division, Department of Internal Medicine, Miaoli General Hospital, Ministry of Health and Welfare, Miaoli, Taiwan; 3 National Yang-Ming University School of Medicine, Taipei, Taiwan; 4 Department of Medical Imaging and Radiological Sciences, I-Shou University, Kaohsiung, Taiwan; 5 Division of Cardiology, Department of Internal Medicine, E-Da Hospital, Kaohsiung, Taiwan; 6 Institute of Clinical Medicine and Cardiovascular Research Center, National Yang-Ming University, Taipei, Taiwan; 7 Divison of Cardiology, Department of Medicine, Taipei Veterans General Hospital, Taipei, Taiwan; 8 Division of Cardiology, Heart Center, Cheng-Hsin General Hospital, and School of Medicine, National Yang-Ming University, Taipei, Taiwan; 9 Division of Cardiology, Department of Internal Medicine, Kaohsiung Medical University Hospital and Kaohsiung Medical University, Kaohsiung, Taiwan; 10 Division of Cardiovascular Medicine, China Medical University Hospital, Taichung, Taiwan; 11 Graduate Institute of Biomedical Sciences, China Medical University, Taichung, Taiwan; 12 Department of Cardiology, Buddhist Tzu-Chi General Hospital, Tzu-Chi University, Hualien, Taiwan; 13 Cardiovascular Division, Department of Internal Medicine, MacKay Memorial Hospital, Mackay Medical College, New Taipei City, Taiwan; 14 Division of Cardiology, Department of Internal Medicine, National Taiwan University Hospital and National Taiwan University College of Medicine, Taipei, Taiwan; 15 Graduate Institute of Medical Education & Bioethics, College of Medicine, National Taiwan University, Taipei, Taiwan

Correction to: *Scientific Reports* 10.1038/s41598-018-32210-x, published online 26 September 2018

The original version of this Article contained typographical errors, where the unit ‘ng/ml’ was incorrectly given as ‘pg/ml’. As a result, in the Abstract,

“The cut-off value of H-FABP, 4.143 pg/mL, was determined using receiver operating characteristic curves.”

now reads:

“The cut-off value of H-FABP, 4.143 ng/mL, was determined using receiver operating characteristic curves.”

In the Results section under subheading ‘Patients’,

“The cut-off value of H-FABP (4.143 pg/mL) was determined by receiver operating characteristic curves (ROC) curve analysis (Fig. 1) between the patients with and without CV events from the blood sample obtained at enrollment.”

now reads:

“The cut-off value of H-FABP (4.143 ng/mL) was determined by receiver operating characteristic curves (ROC) curve analysis (Fig. 1) between the patients with and without CV events from the blood sample obtained at enrollment.”

In the Discussion section,

“This study is the first prospective cohort study to demonstrate that a higher serum H-FABP level (≧4.143 pg/mL) is an independent predictor for CV events, particularly for cardio- and cerebrovascular death and acute heart failure-related hospitalizations in patients with SCHD.”

now reads:

“This study is the first prospective cohort study to demonstrate that a higher serum H-FABP level (≧4.143 ng/mL) is an independent predictor for CV events, particularly for cardio- and cerebrovascular death and acute heart failure-related hospitalizations in patients with SCHD.”

In Table 2, the column headings ‘H-FABP < 4.143 pg/mL’ and ‘H-FABP ≧ 4.143 pg/mL’ have been corrected to ‘H-FABP < 4.143 ng/mL’ and ‘H-FABP ≧ 4.143 ng/mL’, respectively.

In Table 3, the column headings ‘H-FABP <4.143pg/mL, (n=843)’ and ‘H-FABP ≧4.143pg/mL, (n=228)’ have been corrected to ‘H-FABP <4.143ng/mL, (n=843)’ and ‘H-FABP ≧4.143ng/mL, (n=228)’, respectively.

In the legend of Figure 2,

“Kaplan–Meier survival curves analysis showing total cardiovascular (CV) event-free rate (**a**), CV or cerebrovascular death-free rate (**b**), acute heart failure hospitalization-free rate (**c**), and total CV event-free rate except for angina-related hospitalization (**d**) in patients with serum H-FABP ≧4.143 pg/mL and H-FABP <4.143 pg/mL (all p < 0.001).”

now reads:

“Kaplan–Meier survival curves analysis showing total cardiovascular (CV) event-free rate (**a**), CV or cerebrovascular death-free rate (**b**), acute heart failure hospitalization-free rate (**c**), and total CV event-free rate except for angina-related hospitalization (**d**) in patients with serum H-FABP ≧4.143 ng/mL and H-FABP <4.143 ng/mL (all p < 0.001).”

In addition, the original version of this Article contained typographical errors, where the unit ‘g/dL’ was incorrectly given as ‘mg/dL’. As a result, in Table 2, the row heading ‘Hemoglobin, mg/dL’ has been corrected to ‘Hemoglobin, g/dL’.

These errors have now been corrected in the HTML and PDF versions of the Article.

