# Supplementary material for: The prognostic significance of heart-type fatty acid binding protein in patients with stable coronary heart disease
Source: Sci Rep. 2018 Sep 26;8:14410. doi: 10.1038/s41598-018-32210-x (PMC6158177; doi:10.1038/s41598-018-32210-x)
Supplement: Supplementary file 1 — Supplementary Information [file 41598_2018_32210_MOESM1_ESM.docx]

Supplementary information of the article:

**The prognostic significance of heart-type fatty acid binding protein in patients with stable coronary heart disease**

Sing-Kong Ho, Yen-Wen Wu, Wei-Kung Tseng, Hsin-Bang Leu, Wei-Hsian Yin, Tsung-Hsien Lin, Kuan-Cheng Chang, Ji-Hung Wang, Hung-I Yeh, Chau-Chung Wu, Jaw-Wen Chen, and on behalf of the National Taiwan Biosignature Research Investigators.

**Consortium members of National Taiwan Biosignature Research**

Principle investigator:

Jaw-Wen Chen^1^

Co-principle investigators:

Yen-Wen Wu^2^, Wei-Kung Tseng^3^, Hsin-Bang Leu^1^, Wei-Hsian Yin^4^, Tsung-Hsien Lin^5^, Kuan-Cheng Chang^6^, Ji-Hung Wang^7^, Hung-I Yeh^8^, Chau-Chung Wu^9^

^1^ Divison of Cardiology, Department of Medicine, Taipei Veterans General Hospital, Taipei, Taiwan

^2^ Cardiology Division of Cardiovascular Medical Center, Far Eastern Memorial Hospital, New Taipei City, Taiwan

^3^ Division of Cardiology, Department of Internal Medicine, E-Da Hospital, Kaohsiung, Taiwan

^4^ Division of Cardiology, Heart Center, Cheng-Hsin General Hospital, and School of Medicine, National Yang-Ming University, Taipei, Taiwan

^5^ Division of Cardiology, Department of Internal Medicine, Kaohsiung Medical University Hospital and Kaohsiung Medical University, Kaohsiung, Taiwan

^6^ Division of Cardiovascular Medicine, China Medical University Hospital, Taichung, Taiwan

^7^ Department of Cardiology, Buddhist Tzu-Chi General Hospital, Tzu-Chi University, Hualien, Taiwan

^8^ Cardiovascular Division, Department of Internal Medicine, MacKay Memorial Hospital, Mackay Medical College, New Taipei City, Taiwan

^9^ Division of Cardiology, Department of Internal Medicine, National Taiwan University Hospital and National Taiwan University College of Medicine, Taipei, Taiwan
